# Molecular Characterization of Genital and Extragenital Lesions With the PlexPCR VHS Assay in Patients Diagnosed With Syphilis

**DOI:** 10.1093/ofid/ofad483

**Published:** 2023-09-26

**Authors:** Maria Eguiluz, Jazmin Qquellon, Silver K Vargas, Michael Reyes-Diaz, Kelika A Konda, Carlos F Caceres, Jeffrey D Klausner

**Affiliations:** Center for Interdisciplinary Studies in Sexuality, AIDS and Society, Universidad Peruana Cayetano Heredia, Lima, Peru; Center for Interdisciplinary Studies in Sexuality, AIDS and Society, Universidad Peruana Cayetano Heredia, Lima, Peru; Center for Interdisciplinary Studies in Sexuality, AIDS and Society, Universidad Peruana Cayetano Heredia, Lima, Peru; School of Public Health and Administration, Universidad Peruana Cayetano Heredia, Lima, Peru; Center for Interdisciplinary Studies in Sexuality, AIDS and Society, Universidad Peruana Cayetano Heredia, Lima, Peru; Center for Interdisciplinary Studies in Sexuality, AIDS and Society, Universidad Peruana Cayetano Heredia, Lima, Peru; Department of Population and Public Health Sciences, Keck School of Medicine, University of Southern California, Los Angeles, California, USA; Center for Interdisciplinary Studies in Sexuality, AIDS and Society, Universidad Peruana Cayetano Heredia, Lima, Peru; Department of Population and Public Health Sciences, Keck School of Medicine, University of Southern California, Los Angeles, California, USA

**Keywords:** clinical diagnosis, genital lesions, PlexPCR VHS assay, syphilis

## Abstract

**Background:**

Syphilis diagnosis relies on immunologic markers and clinical protocols. However, syphilitic lesions can be confused with other genital ulcer diseases.

**Methods:**

Using a PlexPCR VHS assay, we analyzed lesion DNA samples from 87 individuals who were clinically diagnosed with early syphilis infection and had at least 1 positive serologic test result. DNA was detected by the PlexPCR VHS multiplex assay and β-globin genes.

**Results:**

Among the participants, 99% (86/87) had a positive rapid treponemal test result. DNA was successfully detected in 91% (79/87) of the lesion samples. PlexPCR VHS identified 5 herpes simplex virus (HSV)/*Treponema pallidum* coinfections (2 HSV-1 and 3 HSV-2), only *T pallidum* DNA in 62% (49/79), and only HSV-2 in 12.7% (10/79). While 19% (15/79) were negative for all pathogens, none were varicella zoster virus positive. The PlexPCR VHS had 68.4% agreement with the clinical diagnosis.

**Conclusions:**

Since the PlexPCR VHS detects multiple organisms simultaneously, it can help to confirm actual syphilis and identify other pathogen coinfections or the pathogen causing the ulcer.

## Graphical Abstract

**Graphical Abstract:**
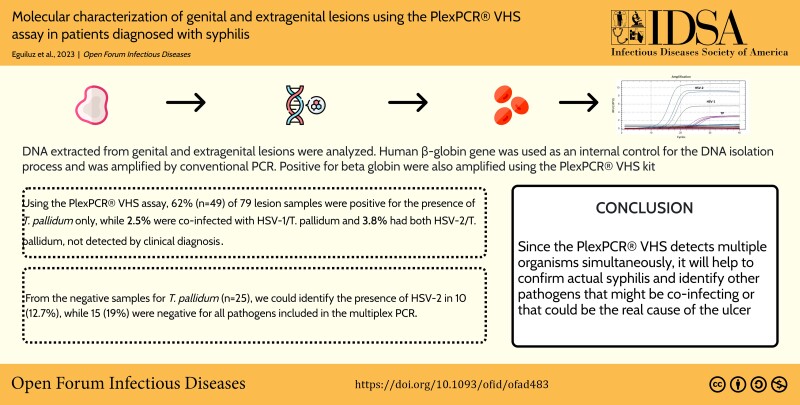
This graphical abstract is also available at Tidbit: https://tidbitapp.io/tidbits/molecular-characterization-of-genital-lesions-using-the-plexpcr-vhs-assay-in-patients-diagnosed-with-syphilis-40a3e9ca-015d-450c-8c5c-945e0a551047

Syphilis is a sexually transmitted infection (STI) that remains a considerable public health problem and has serious medical consequences if left untreated [1]. It is caused by the bacterium *Treponema pallidum* sub *pallidum* (*T pallidum*) and is a leading cause of preventable infant mortality, surpassing HIV infection globally [2, 3]. Syphilis is diagnosed by combining clinical examination for signs and symptoms with 2 types of serologic tests: a nontreponemal test, such as rapid plasma reagin (RPR), and a treponemal test, such as *T pallidum* particle agglutination (TPPA) or a *T pallidum* rapid test. The primary stage of syphilis infection is usually characterized by the presence of a single painless ulcer (chancre) at the infection site, but atypical presentations with multiple and/or painful lesions have also been reported, especially among people living with HIV [4, 5].

Other STIs, such as herpes simplex virus type 1 (HSV-1) and type 2 (HSV-2), produce genital ulcers and extragenital skin lesions. Those lesions can occur alone or as coinfections with syphilis; recently, varicella zoster virus (VZV) has also been reported as a causal agent of genital lesions [5–8]. All of those infectious agents, including *T pallidum*, can be differentially detected by molecular techniques. Real-time polymerase chain reaction (PCR) can detect each agent by the presence of its genetic material, and this procedure can be performed with a multiplex PCR [9].

The most widely used approach to address STIs is syndromic management; this is especially true in resource-limited settings where laboratory diagnosis is not available or is hard to access [10]. This type of management, based solely on signs and symptoms, is an unreliable means for distinguishing genital ulcer diseases (GUDs) and is especially complicated in atypical presentations, which are more frequent among people living with HIV. As in the context of this study, clinical diagnosis can be supported by serologic testing, although this has limitations, such as low sensitivity in early infection and the inability to confirm *T pallidum* presence or discern coinfections; all of which can lead to poor clinical management [11–13]. For syphilis diagnosis, molecular detection of *T pallidum* is not routinely performed in patients with GUD. The use of molecular assays would help people living with HIV, whose primary syphilis may present with more, larger, or deeper ulcers or concomitant secondary symptoms [14, 15]. Molecular tests can confirm the presence of the spirochete *T pallidum* bacteria not discernible by clinical evaluation alone or by serologic tests during the early primary stage of syphilis infection [16]. Multiplex assays can also help clinicians to detect other pathogens in the lesion to guide the appropriate treatment [17].

In this study we assessed the diagnostic agreement of the PlexPCR VHS assay with clinical diagnosis confirmed by serologic tests using DNA isolated from lesion swabs from patients diagnosed with early syphilis.

## METHODS

### Study Sites and Population

Samples were from participants recruited at 5 sexual health clinics in Peru: 3 in Metropolitan Lima, the capital city; 1 in the Callao region; and 1 in Pucallpa, located in the eastern part of the country. Recruitment took place from May 2019 to August 2021 as part of an ongoing cohort study (PICASSO 2) [18]. Patients had to be at least 18 years old, with genital or extragenital lesions diagnosed with active syphilis by clinical examination and with at least 1 positive serologic test result. The study protocol was approved by the institutional review board at Universidad Peruana Cayetano Heredia (SIDISI 103093).

### Clinical Diagnosis of Syphilis

Per the guidelines of the Centers for Disease Control and Prevention [1], syphilis was clinically diagnosed by combining serologic testing and clinical history, including clinical characteristics and/or the patient's sexual history. Syphilis diagnostic testing was based on the reverse algorithm and began with a treponemal-specific antibody rapid test (Determine Syphilis TP; Abbott) with reflex RPR testing (RPR slide test; Wiener Laboratorios SAIC) performed at the enrollment site on the day of diagnosis and enrollment. Participants received syphilis treatment according to the clinical and serologic testing available at the enrollment site and the STI treatment guidelines of the Centers for Disease Control and Prevention: early syphilis cases were treated with a single dose of intramuscular benzathine penicillin, and late syphilis or syphilis of unknown duration was treated with 3 doses. Participants were also asked to provide, and some consented to staff taking pictures of their lesions for future use. Then serum aliquots were transported on ice to the Laboratory of Sexual Health, located at Universidad Peruana Cayetano Heredia, Lima, for confirmatory testing based on the TPPA (SERODIA-TP-PA; Fujirebio). Clinical and serologic responses were monitored 1 month after treatment among all participants of the PICASSO 2 study and quarterly to 12 months among participants with or without known information about *T pallidum* past infection.

### DNA Samples

DNA extracted from genital and extragenital lesions was analyzed. In the case of patients with multiple-lesion samples, 1 isolated DNA sample was selected. DNA was extracted from 500 μL of swab exudate resuspended in lysis buffer (1M Tris, pH 8.0, 0.5M EDTA, 10% SDS) with the QIAamp DNA Mini Kit (Qiagen) according to the manufacturer's instructions and preserved at −80 °C until use. The human β-globin gene was used as an internal control for the DNA isolation process. The β-globin gene was amplified by conventional PCR according to methods described in the literature [19]. Samples that failed to amplify were retested. If a second negative result for β-globin amplification was obtained, the sample was excluded from further analysis. The PCR products were electrophoresed on a 1% agarose gel with a 100-bp DNA ladder (Invitrogen) and visualized by ultraviolet transillumination of the ethidium bromide–stained gel. DNA samples detected as positive for β-globin were used for the PlexPCR VHS assay.

### PlexPCR VHS Qualitative Real-time PCR Assay

The PlexPCR VHS assay (SpeeDx Inc) uses a novel real-time PCR technology to amplify and detect HSV-1, HSV-2, *T pallidum*, and VZV nucleic acid. It includes an internal control to validate the DNA extraction procedure and monitor amplification efficiency, but this was not used in our study as the DNA samples were already isolated, as described earlier. The PlexPCR VHS assay was performed following the manufacturer's instructions; briefly, 1 μL of the VHS mix (20×), containing whole oligonucleotides for amplification, was combined with 10 μL of Plex Mastermix (2×) and nuclease-free water to complete 20 μL of final volume in a CFX-96 RealTime PCR System (Biorad, USA). Results were analyzed using the PlexPCR VHS (CFX) analysis software (version 1.0).

### Data Analysis

For statistical analysis, medians and proportions for descriptive variables and distribution of infectious agents identified in lesions were calculated. To evaluate the performance of the PlexPCR VHS test, we estimated the percentage of positive agreement with clinical diagnosis as the reference comparator (defined as the percentage of positive *T pallidum* PCR results among all clinically identified syphilis cases) and calculated the exact binomial 95% CI. We assessed the frequency of *T pallidum*, HSV, and VZV using the PlexPCR VHS assay. Then, we stratified by *T pallidum* PlexPCR positivity to evaluate any coinfections with HSV and/or VZV in *T pallidum–*positive samples and to identify which agents were present in *T pallidum*–negative samples. We then evaluated the distribution of infectious agents according to lesion characteristics and used the Fisher exact test to compare lesion number, location, and type with HIV status. All statistical analyses were conducted with Stata version 14 (StataCorp).

## RESULTS

DNA isolated from lesion swabs was collected from 87 individuals clinically diagnosed with syphilis based on the presence of lesion and serology. From the 87 patients, we successfully amplified the human β-globin gene fragment from 79 samples from as many patients. The median age of participants evaluated was 33 years (IQR, 26–40), and 73.4% were MSM (58/79). Most samples evaluated were from patients diagnosed with primary syphilis (n = 72, 91.1%). Around half of our population was HIV infected (n = 42, 53.2%). Almost all participants had a positive rapid antibody treponemal test result except for 1; however, that person was RPR reactive and TPPA positive. Among the participants, 96.2% had a positive TPPA test result, 27.9% had low RPR titer (≤1:4), and 10.1% were RPR nonreactive (Table 1).

**Table 1.** Characteristics of STI Clinic Users Diagnosed With Early Syphilis (N = 79)

Variable	No. (%)
Age^a^	33 (26–40)
Sexual orientation	
Heterosexual	4 (5.1)
Gay/homosexual	58 (73.4)
Bisexual/other	17 (21.5)
Primary syphilis	
No	7 (8.9)
Yes	72 (91.1)
History of syphilis	
No	11 (13.9)
Yes	13 (16.5)
Unknown	55 (69.6)
HIV status	
Negative	37 (46.8)
Positive	42 (53.2)
Ulcer location	
Genital	58 (73.4)
Perianal	14 (17.7)
Others	7 (8.9)
Lesion type	
Painful	23 (29.1)
Painless	56 (70.9)
No. of lesions	
Single	41 (51.9)
Multiple	35 (44.3)
Unknown	3 (3.8)
Rapid Test (Determine Syphilis TP; Abbott)	
Positive	78 (98.7)
Negative	1 (1.3)
RPR	
Nonreactive	8 (10.1)
Reactive ≤ 1:4	22 (27.9)
Reactive ≥ 1:8	49 (62.0)
TPPA	
Negative	3 (3.8)
Positive	76 (96.2)
PlexPCR VHS^b^	
Only *T pallidum*	49 (62.0)
*T pallidum* and HSV-1	2 (2.5)
*T pallidum* and HSV-2	3 (3.8)
Only HSV-2	10 (12.7)
Negative for all	15 (19.0)

Abbreviations: HSV-1, herpes simplex virus type 1; HSV-2, herpes simplex virus type 2; RPR, rapid plasma reagin; STI, sexually transmitted infection; *T pallidum*, *Treponema pallidum*; TPPA, *Treponema pallidum* particle agglutination.

^a^Median (p25–p75).

^b^No positives were detected for varicella zoster virus or only HSV-1.

Lesion swabs were collected from the genital area (n = 58, 73.4%) and perianal area (n = 14, 17.7%), and the remainder were from other parts of the body (n = 7, 8.9%). Extragenital lesions were more common in men infected with HIV (35.7%) than in men without HIV infection (16.2%), but this did not reach statistical significance (*P* = .074). Most lesions were classified as painless, but 29.1% were painful. Most individuals had just 1 lesion (n = 41, 51.9%), while 35 (44.3%) had multiple lesions (Table 1). The proportion of individuals with multiple lesions was significantly higher when the lesions were reported as painful vs painless (68.2% vs 37.0%, *P* = .022).

**Figure 1.**
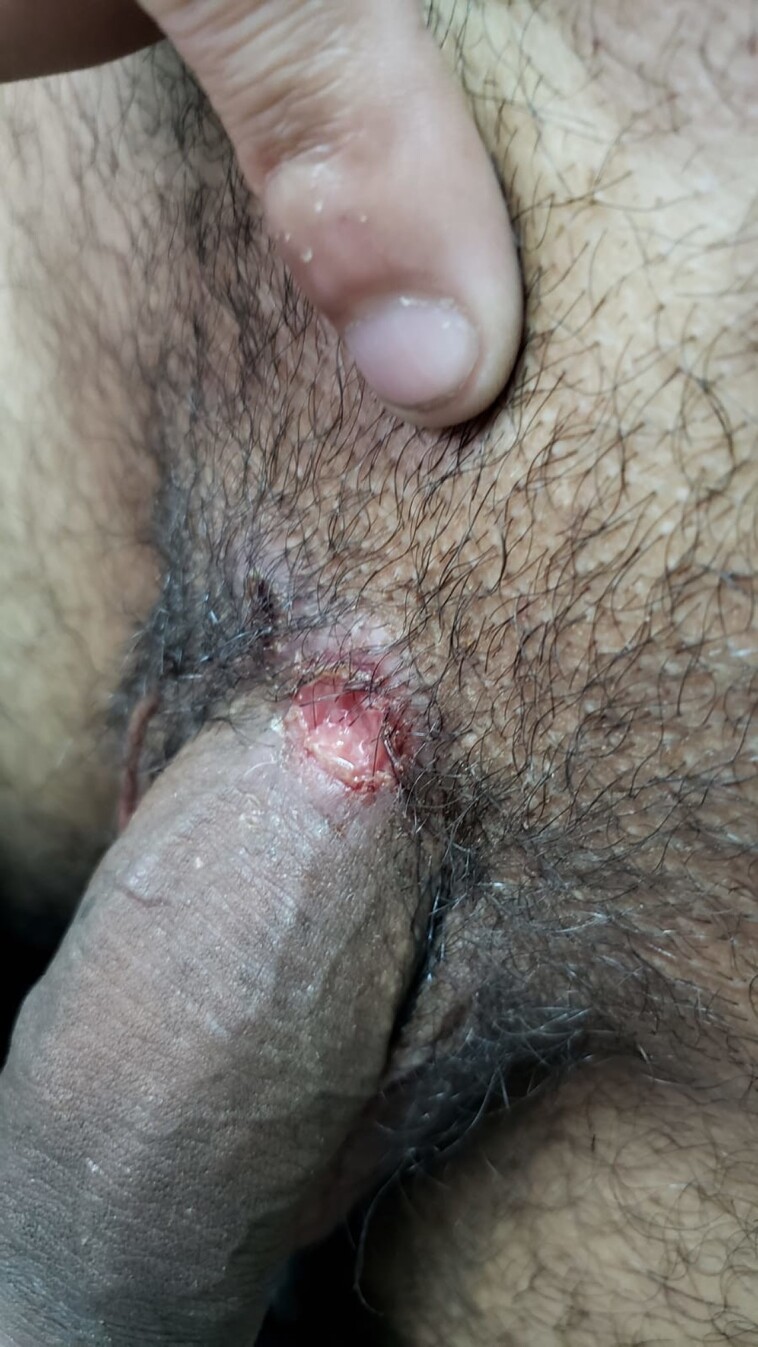
A single painless lesion at the base of the penis with the coinfection of *Treponema pallidum* and herpes simplex virus type 1.

**Figure 2.**
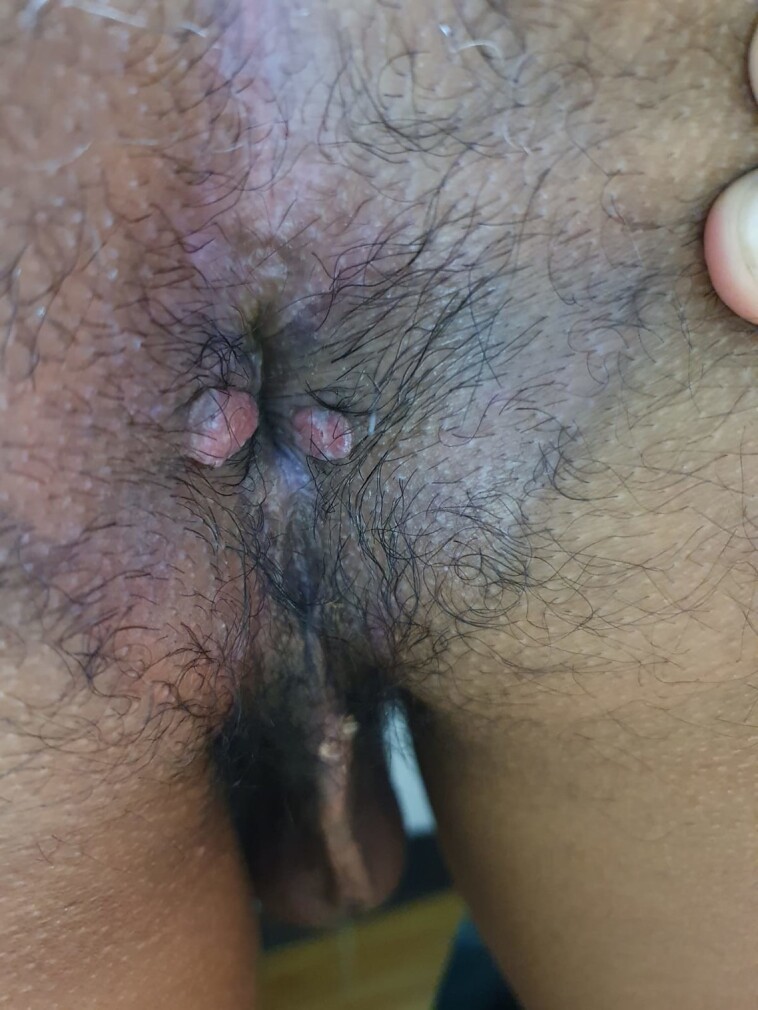
Two painful perianal lesions with the coinfection of *Treponema pallidum* and herpes simplex virus type 2.

According to the PlexPCR VHS assay, 62% (n = 49) of 79 lesion samples were positive for the presence of *T pallidum* only, while 2 samples (2.5%) were coinfected with HSV-1/*T pallidum* and 3 samples (3.8%) had HSV-2/*T pallidum* (Table 1). Among the samples where only *T pallidum* was detected, most were diagnosed as primary syphilis and had a single painless lesion located in the genital area (Table 2). Figure 1 shows an example of a single painless lesion at the base of the penis with unknown previous *T pallidum* exposure. The 2 HSV-1/*T pallidum* coinfection lesions were located in the genital area: both were clinically diagnosed as primary syphilis lesions and 1 was painless (Table 2). Both patients were treated with 1 dose of penicillin. At 4 weeks, 1 patient responded to treatment; specifically, the RPR titer decreased 4-fold and the lesion resolved. The 3 HSV-2/*T pallidum* coinfected samples were also diagnosed as primary syphilis. One was treated with 3 doses of penicillin while the others were treated with 1 dose of penicillin; 2 had a 4-fold titer reduction after 4 weeks. Two were described as multiple painless lesions located around the genital area; however, 1 presented with 2 painful perianal lesions (Table 2, Figure 2). We did not detect any lesions with VZV.

**Table 2.** Characteristics of STI Clinic Users With PlexPCR VHS–Positive Specimens (n = 64)

	No. (%)			
	Only TP (n = 49)	TP and HSV-1 (n= 2)	TP and HSV-2 (n = 3)	Only HSV-2 (n = 10)
Syphilis stage				
Primary	44 (89.8)	2 (100.0)	3 (100.0)	9 (90.0)
Secondary	5 (10.2)			1 (10.0)
Lesion type				
Painful	14 (28.6)	1 (50.0)	1 (33.3)	2 (20.0)
Painless	35 (71.4)	1 (50.0)	2 (66.7)	8 (80.0)
Lesion location				
Genital	42 (85.7)	2 (100.0)	2 (66.7)	5 (100.0)
Perianal	4 (8.2)		1(33.3)	5 (100.0)
Perineum	2 (4.1)			
Mucous patch	1 (2.0)			
No. of lesions				
Single	24 (49.0)	2 (100.0)	1 (33.3)	6 (60.0)
Multiple	24 (49.0)		2 (66.7)	4 (40.0)
Unknown	1 (2.0)			

Abbreviations: HSV-1, herpes simplex virus type 1; HSV-2, herpes simplex virus type 2; STI, sexually transmitted infection; TP, *Treponema pallidum*.

From the 31% (n = 25) of samples that were negative for *T pallidum*, we could identify the presence of HSV-2 in 10 (12.7%), while 15 (19%) were negative for all pathogens included in the multiplex PCR (Table 1). For HSV-2–positive cases, 4 of 10 patients were treated with 3 doses of penicillin. From this group, 3 reported a prior syphilis infection while the other did not have prior syphilis. The remaining patients (7/10) had no prior syphilis infection and were treated with 1 dose of penicillin. For the patients with *T pallidum*–negative samples, most (14/15) were diagnosed as having primary syphilis and treated with 1 dose of penicillin. Among these 14 patients, 5 reported a prior syphilis infection while the other 9 had no prior infection. The remaining patient (1/15) was diagnosed with suspected tertiary syphilis, as this person had prior syphilis and was treated with 3 doses of penicillin.

PlexPCR VHS assay detected *T pallidum* DNA in 68% (n = 38) of painless lesions and 70% (n = 16) of painful lesions. For the HSV-2–positive lesions, the majority (70%, 7/10) were painless. Among *T pallidum*/HSV coinfections, 3 samples were from painless lesions and 2 from painful lesions (Table 2).

According to the clinical diagnosis, all individuals who provided lesion samples (N = 79) were diagnosed with syphilis. However, according to the PlexPCR VHS, just 54 of those were positive for *T pallidum*, either by itself or in coinfection with HSV. Therefore, the positive agreement between the diagnostic methods of syphilis was 68.4% (95% CI, 56.9%–78.4%; Table 3). Among the 79 clinically diagnosed patients, 9 did not have a positive serologic test result: 2 samples were negative for RPR and TPPA; 1 was negative for TPPA only; and 6 were RPR nonreactive. Furthermore, the PlexPCR VHS assay identified *T pallidum* DNA in 6 samples; HSV-2 DNA was detected in 2 samples; and the last was negative for all pathogens.

**Table 3.** Positive Agreement of PlexPCR VHS *Treponema pallidum*–Positive Samples vs Clinical Diagnosis

	No.	Positive Agreement (95% CI), %
PlexPCR VHS	79	68.4 (56.9–78.4)
Positive	54	
Negative	25	

Reference test: clinical diagnosis.

## DISCUSSION

Among patients in the STI clinic who were clinically diagnosed with early syphilis and presented with genital lesions, two-thirds had detectable *T pallidum* DNA in their lesion sample using the PlexPCR VHS assay. A few were coinfected with either HSV-1 or HSV-2, and we were able to detect the presence of HSV-2 in some of the samples that were negative for *T pallidum* DNA. Although other studies have reported high positive agreement (94.6%–100%) in panels of genital samples previously tested by in-house laboratory-based assays [20], our lower positive agreement could be due to the use of clinical diagnosis (supported by serologic testing) as the reference for comparison.

Considering the simultaneous detection of multiple DNA targets for different organisms, we were able to detect some patients diagnosed with syphilis who were coinfected with HSV (6.3%). Furthermore, of the lesions without detectable *T pallidum* DNA, 10 of 25 (40%) were positive for HSV-2. All of these patients were treated with penicillin and, as far we know, did not receive antiviral medication. Some of these patients could have benefited from antiviral medication, as early treatment is reported to reduce lesion healing time and therefore reduce the risk of HSV transmission [21] and HIV acquisition [22]. Another study implemented a commercial molecular test for the diagnosis of syphilis and compared it with a final diagnosis based on clinical examination, serologic tests, and an in-house nested PCR detection assay. For all lesions negative for syphilis, the authors found that 14% of samples were positive for HSV-1, 20% for HSV-2, and 0.6% for lymphogranuloma venereum (LGV) [23]. These results demonstrate the utility of tests that can detect multiple organisms in genital ulcers and thus can complement clinical examination in settings where molecular assays with timely results are available, allowing patients to be treated appropriately by preventing the overuse of antibiotics and reducing the demand of benzathine penicillin, which has generated a shortage of this antibiotic in countries across the resource spectrum (ie, high-, low-, and middle-income countries) [24, 25].

We found that 19% of all samples were negative for the 4 organisms tested. A similar frequency was reported in South Africa and Malawi [26, 27]. One of the reasons could be that no tests have been performed for other etiologies that cause ulcers, including trauma, streptococcus or staphylococcus, or other STIs (eg, *Haemophilus ducreyi*, donovanosis, or LGV). In Peru, previous studies have cited a prevalence of *H ducreyi* in 27% of female sex workers [28] and 5% of STI clinic attendees with GUD [29]. Studies have found few cases of donovanosis (before 2010) [30] and the absence of LGV among MSM [31]. In addition to the lack of testing for other potential etiologies, negative samples could be due to a late phase of primary syphilis with a low bacterial load in the sample or to possible PCR inhibition or competition in the case of coinfections that could not be excluded since the PlexPCR VHS internal control was not used. These reasons could explain the negative results in samples with adequate human DNA, as validated by the presence of human β-globin [14, 32].

Using the PlexPCR VHS assay, we could detect *T pallidum* DNA in 2 of the 3 TPPA-negative samples and in 6 of the 8 RPR-nonreactive samples. Previous studies compared multiplex PCR assays with serologic testing for syphilis and detected that their concordance was around 50%, although molecular testing detected *T pallidum* DNA in patients who were serologically negative (<6.5%) [14, 33]. While serologic tests lack sensitivity in the very early phase of primary syphilis [34], PCR protocols are highly sensitive in this phase [16]. Multiplex PCR allowed the identification of primary syphilis prior to the development of serologically detectable antibodies, which has important implications for early treatment [35, 36].

Primary syphilis is characterized by the presence of a chancre, which has been classically described as a single indurated painless ulcer at the site of *T pallidum* inoculation. However, ulcers can be atypical [32]. In our study, 28.6% (n = 35) of *T pallidum*–positive DNA samples identified by the PlexPCR VHS assay (n = 49) were in lesions described as painful by the individuals. Our results agree with Towns et al [5] and Richardson et al [37], who found that the frequency of painful syphilitic lesions in the absence of herpes is between 29% and 49%. Yet, painful lesions can suggest coinfection or may be misdiagnosed since other GUD, such as herpes, is characterized by the presence of multiple painful lesions [38]. This is similar to our finding that the PlexPCR VHS assay identified *T pallidum*/HSV-1 and *T pallidum*/HSV-2 coinfections in 6.3% of the samples and HSV-2 in 12.7%.

Syndromic management is an inexpensive method for the clinical management of symptomatic STIs [39], which recommends treatment for all possible causative pathogens of a lesion. However, clinicians often provide specific treatment for just 1 pathogen. This may be increased due to the serologic evidence provided by syphilis testing, whereas no serologic testing is typically used for HSV in these settings. Following international syndromic guidelines is particularly relevant in the case of coinfections or atypical chancre presentation, which is not infrequent as evident in our results. In both cases, mistreatment could favor the perpetuation of the illness or indirectly increase antimicrobial resistance because of the unnecessary use of antibiotics. Additionally, our results could help to adapt STI treatment guidelines for an efficient treatment and highlight the sensitivity and versatility of molecular techniques such as PlexPCR VHS, which could help in the characterization of GUD.

There are several important limitations to our findings. Our analysis is limited to samples from patients who had lesions and were clinically diagnosed with syphilis. Clinical diagnosis confirmed by at least 1 serologic test result was used as a reference, although it usually fails when there are atypical manifestations of the lesions. Additionally, as we used stored DNA samples, the internal control from the PlexPCR VHS assay could not be added at the beginning of DNA isolation process to be used as a reference for real-time PCR amplification later on in the experiment. Instead, we used the human β-globin gene to validate the DNA isolation process. For validation, we identified a few negative samples (n = 8), which were potentially taken poorly by the clinicians or had a low cell load. We could not re-extract the DNA from those negative samples for either β-globin or the PlexPCR VHS assay due to the lack of sufficient lesion sample volume and because the approved protocol indicated taking a sample from the lesion at only the beginning of the study.

Our findings could promote clinicians to correctly use or adapt the syndromic management guidelines to address local patterns of infection. Additionally, multiplex PCR molecular techniques, whenever the infrastructure exists, could be added to provide a specific etiologic diagnosis or used for GUD prevalence studies. While multiple PCR techniques would help clinicians with accurate etiologic identification, the cost is prohibitive in many settings. Clinicians should consider the atypical presentation of syphilitic lesions that may be identified as herpes, which can lead to mistreatment, or for coinfection cases that require treatment for both diseases. Additionally, accurate identification and treatment of GUD is crucial since untreated infections are known risk factors for HIV infection [32]. Finally, our findings should ideally be validated by conducting field and prospective studies in multiple sites in Peru and expanding the population study for all patients with any genital and nongenital ulcers for evaluating the etiology of GUD.

## Notes

***Acknowledgments.*** We acknowledge members of the field team (interviewers, counselors, laboratory technicians, and team leaders) who collected the data and conducted syphilis testing and to all the study participants who contributed their time, information, and samples. We are grateful to Dr. Lorenzo Giacani for his assistance in writing the manuscript.

***Author contributions.*** M. E.: conceptualization, methodology, writing–original draft, writing–review and editing. J. Q.: formal analysis, writing–original draft, writing–review and editing. S. K. V.: methodology, supervision, writing–review and editing. M. R.-D.: investigation, writing–review and editing. K. A. K.: conceptualization, funding acquisition, writing–review and editing. C. F. C: conceptualization, funding acquisition, writing–review and editing. J. D. K.: conceptualization, funding acquisition, writing–review and editing.

***Patient consent statement.*** Written consent was obtained from all patients who participated in this study. The study design was approved by the Universidad Peruana Cayetano Heredia Institutional Review Board (SIDISI 103093).

***Disclaimer.*** Speedx did not have any role in the study design, implementation, or data analysis.

***Financial support*.** This work was supported by Speedx Pty Ltd; and by the National Institutes of Health (1R01AI39265-01) for PICASSO 2, an ongoing project in Lima, Peru.

## References

[1] Centers for Disease Control and Prevention . STI treatment guidelines. 2021. Available at: https://www.cdc.gov/std/treatment-guidelines/default.htm. Accessed 22 May 2022.

[2] Bhutta ZA , Yakoob MY, Lawn JE, et al Stillbirths: what difference can we make and at what cost? Lancet 2011; 377:1523–38. 21496906 10.1016/S0140-6736(10)62269-6

[3] Stamm LV . Syphilis: re-emergence of an old foe. Microb Cell 2016; 3:363–70. 28357375 10.15698/mic2016.09.523 PMC5354565

[4] Tiecco G , Degli Antoni M, Storti S, et al A 2021 update on syphilis: taking stock from pathogenesis to vaccines. Pathogens 2021; 10:1364. 34832520 10.3390/pathogens10111364 PMC8620723

[5] Towns JM , Leslie DE, Denham I, Azzato F, Fairley CK, Chen M. Painful and multiple anogenital lesions are common in men with *Treponema pallidum* PCR-positive primary syphilis without herpes simplex virus coinfection: a cross-sectional clinic-based study. Sex Transm Infect 2016; 92:110–5. 26378262 10.1136/sextrans-2015-052219

[6] Birch CJ , Druce JD, Catton MC, MacGregor L, Read T. Detection of varicella zoster virus in genital specimens using a multiplex polymerase chain reaction. Sex Transm Infect 2003; 79:298–300. 12902579 10.1136/sti.79.4.298 PMC1744717

[7] LeGoff J , Péré H, Bélec L. Diagnosis of genital herpes simplex virus infection in the clinical laboratory. Virol J 2014; 11:83. 24885431 10.1186/1743-422X-11-83 PMC4032358

[8] Magdaleno-Tapial J , Hernández-Bel P, Ortiz-Salvador JM, et al Genital herpes zoster: a rare location that can mimic genital herpes. Sex Transm Dis 2022; 49:e34–6. 33993159 10.1097/OLQ.0000000000001465

[9] Nikolic D , Kohn D, Yen-Lieberman B, Procop GW. Detection of herpes simplex virus and varicella-zoster virus by traditional and multiplex molecular methods. Am J Clin Pathol 2019; 151:122–6. 30239569 10.1093/ajcp/aqy111

[10] World Health Organization . Guidelines for the management of symptomatic sexually transmitted infections. Available at: https://www.who.int/publications-detail-redirect/9789240024168. Accessed 22 May 2022. 34370424

[11] Shah D , Marfatia Y. Serological tests for syphilis. Indian J Sex Transm Dis 2019; 40:186. 10.4103/ijstd.IJSTD_86_19 PMC6896393 31922115

[12] Prabhakar P , Narayanan P, Deshpande GR, et al Genital ulcer disease in India: etiologies and performance of current syndrome guidelines. Sex Transm Dis 2012; 39:906–10. 23064541 10.1097/OLQ.0b013e3182663e22

[13] Loh AJW , Ting EL, Wi TE, et al The diagnostic accuracy of syndromic management for genital ulcer disease: a systematic review and meta-analysis. Front Med 2022; 8:806605. 10.3389/fmed.2021.806605 PMC8767480 35071282

[14] Gomes Naveca F , Sabidó M, Amaral Pires de Almeida T, et al Etiology of genital ulcer disease in a sexually transmitted infection reference center in Manaus, Brazilian Amazon. PLoS One 2013; 8:e63953. 23704961 10.1371/journal.pone.0063953 PMC3660360

[15] Zetola NM , Klausner JD. Syphilis and HIV infection: an update. Clin Infect Dis 2007; 44:1222–8. 17407043 10.1086/513427

[16] Shukalek CB , Lee B, Fathima S, Chu A, Fonseca K, Somayaji R. Comparative analysis of molecular and serologic testing for primary syphilis: a population-based cohort study. Front Cell Infect Microbiol 2021; 11:579660. 33968792 10.3389/fcimb.2021.579660 PMC8103196

[17] Theel ES , Katz SS, Pillay A. Molecular and direct detection tests for *Treponema pallidum* subspecies *pallidum*: a review of the literature, 1964–2017. Clin Infect Dis 2020; 71:S4–S12. 32578865 10.1093/cid/ciaa176 PMC7312206

[18] Osias E , Hung P, Giacani L, et al Investigation of syphilis immunology and *Treponema pallidum* subsp *pallidum* biology to improve clinical management and design a broadly protective vaccine: study protocol. BMC Infect Dis 2020; 20:444. 32576149 10.1186/s12879-020-05141-0 PMC7309211

[19] Giacani L , Ciccarese G, Puga-Salazar C, et al Enhanced molecular typing of *Treponema pallidum* subspecies *pallidum* strains from 4 Italian hospitals shows geographical differences in strain type heterogeneity, widespread resistance to macrolides, and lack of mutations associated with doxycycline resistance. Sex Transm Dis 2018; 45:237–42. 29465698 10.1097/OLQ.0000000000000741 PMC5847427

[20] Goldstein EJ , Cochrane L, Bone SM, Gunson RN. Performance characteristics of the SpeeDx PlexPCR VHS assay for the molecular detection of herpes simplex virus, varicella zoster virus, and *Treponema pallidum* in lesion swabs. Diagn Microbiol Infect Dis 2021; 99:115221. 33176262 10.1016/j.diagmicrobio.2020.115221

[21] Reichman RC , Badger GJ, Mertz GJ, et al Treatment of recurrent genital herpes simplex infections with oral acyclovir: a controlled trial. JAMA 1984; 251:2103–7. 6368877

[22] Desai DV , Kulkarni SS. Herpes simplex virus: the interplay between HSV, host, and HIV-1. Viral Immunol 2015; 28:546–55. 26331265 10.1089/vim.2015.0012

[23] Grange PA , Jary A, Isnard C, et al Use of a multiplex PCR assay to assess the presence of *Treponema pallidum* in mucocutaneous ulcerations in patients with suspected syphilis. J Clin Microbiol 2021; 59:e01994-20. 33177120 10.1128/JCM.01994-20 PMC8111117

[24] Araujo RS , de Souza ASS, Braga JU. A quem afetou o desabastecimento de penicilina para sífilis no Rio de Janeiro, 2013–2017? Rev Saúde Pública 2020; 54:109. 33146299 10.11606/s1518-8787.2020054002196 PMC7593023

[25] Nurse-Findlay S , Taylor MM, Savage M, et al Shortages of benzathine penicillin for prevention of mother-to-child transmission of syphilis: an evaluation from multi-country surveys and stakeholder interviews. PLoS Med 2017; 14:e1002473. 29281619 10.1371/journal.pmed.1002473 PMC5744908

[26] Paz-Bailey G , Sternberg M, Puren AJ, Steele L, Lewis DA. Determinants of HIV type 1 shedding from genital ulcers among men in South Africa. Clin Infect Dis 2010; 50:1060–7. 20178417 10.1086/651115

[27] Phiri S , Zadrozny S, Weiss HA, et al Etiology of genital ulcer disease and association with HIV infection in Malawi. Sex Transm Dis 2013; 40:923–8. 24220352 10.1097/OLQ.0000000000000051 PMC12814162

[28] Sanchez J , Volquez C, Totten PA, et al The etiology and management of genital ulcers in the Dominican Republic and Peru. Sex Transm Dis 2002; 29:559–67. 12370522 10.1097/00007435-200210000-00001

[29] González-Beiras C , Marks M, Chen CY, Roberts S, Mitjà O. Epidemiology of *Haemophilus ducreyi* infections. Emerg Infect Dis 2016; 22:1–8. 26694983 10.3201/eid2201.150425 PMC4696685

[30] Delgado-Azañero W , Gotuzzo E, Meneses Rivadeneira LV, Lama JR. Granuloma inguinal (Donovanosis) de la encía y regiones submandibulares. Rev Estomatol Herediana 2014; 15:60–6.

[31] Clark JL , Espinosa B, Leon SR, et al Absence of lymphogranuloma venereum infection among high-risk men who have sex with men in Lima, Peru. Int J STD AIDS 2008; 19:427–8. 18595888 10.1258/ijsa.2008.008140 PMC6879098

[32] Klein J , McLaud M, Rogers D. Syphilis on the rise: diagnosis, treatment, and prevention. J Nurse Pract 2015; 11:49–55.

[33] Suntoke TR , Hardick A, Tobian AAR, et al Evaluation of multiplex real-time PCR for detection of *Haemophilus ducreyi*, *Treponema pallidum*, herpes simplex virus type 1 and 2 in the diagnosis of genital ulcer disease in the Rakai District, Uganda. Sex Transm Infect 2008; 85:97–101. 19066198 10.1136/sti.2008.034207 PMC2692240

[34] Creegan L , Bauer HM, Samuel MC, Klausner J, Liska S, Bolan G. An evaluation of the relative sensitivities of the venereal disease research laboratory test and the *Treponema pallidum* particle agglutination test among patients diagnosed with primary syphilis. Sex Transm Dis 2007; 34:1016–8. 18080352

[35] Scott LJ , Gunson RN, Carman WF, Winter AJ. A new multiplex real-time PCR test for HSV1/2 and syphilis: an evaluation of its impact in the laboratory and clinical setting. Sex Transm Infect 2010; 86:537–9. 20643660 10.1136/sti.2009.040451

[36] Zhou L , Gong R, Lu X, Zhang Y, Tang J. Development of a multiplex real-time PCR assay for the detection of *Treponema pallidum*, HCV, HIV-1, and HBV. Jpn J Infect Dis 2015; 68:481–7. 25866106 10.7883/yoken.JJID.2014.416

[37] Richardson D , Kumar P, Jaricha T, Walsh J, Lewis DA. Primary syphilis in men causes painful anogenital lesions and serology is not always helpful. Sex Transm Infect 2020; 96:620. doi: 10.1136/sextrans-2020-054495 32234962

[38] Roett MA . Genital ulcers: differential diagnosis and management. Am Fam Physician 2020; 101:355–61. 32163252

[39] Adams EJ , Garcia PJ, Garnett GP, Edmunds WJ, Holmes KK. The cost-effectiveness of syndromic management in pharmacies in Lima, Peru. Sex Transm Dis 2003; 30:379–87. 12916127 10.1097/00007435-200305000-00002

